# Supplementary material for: Surface Microbiome Profiling of Dental Elevators Using Third‐Generation Sequencing: Implications for Infection Control in Dental Practice
Source: Microbiologyopen. 2025 Dec 8;14(6):e70178. doi: 10.1002/mbo3.70178 (PMC12685768; doi:10.1002/mbo3.70178)
Supplement: Supplementary file 1 — customer_backup. [file MBO3-14-e70178-s001.zip › customer_backup/customer_backup/function_analysis/BugBase/treat/otu_contributions/family/taxa_legend.pdf]

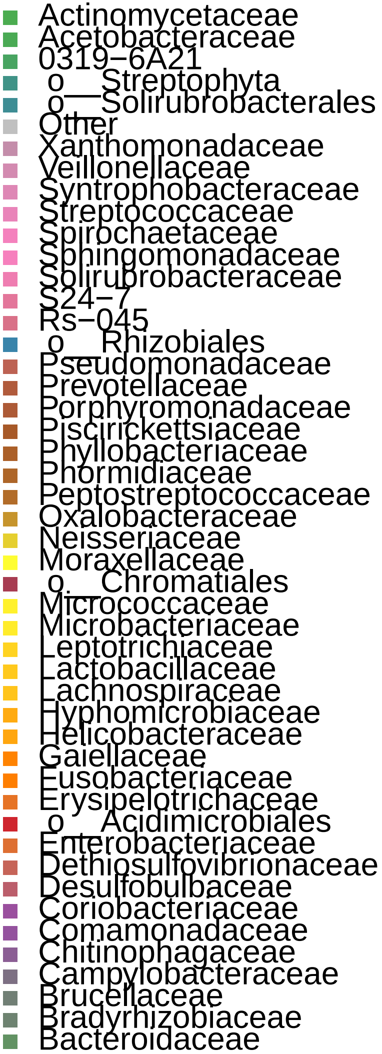
Actinomycetaceae
Acetobacteraceae
0319−6A21
o__Streptophyta
o__Solirubrobacterales
Other
Xanthomonadaceae
Veillonellaceae
Syntrophobacteraceae
Streptococcaceae
Spirochaetaceae
Sphingomonadaceae
Solirubrobacteraceae
S24−7
Rs−045
o__Rhizobiales
Pseudomonadaceae
Prevotellaceae
Porphyromonadaceae
Piscirickettsiaceae
Phyllobacteriaceae
Phormidiaceae
Peptostreptococcaceae
Oxalobacteraceae
Neisseriaceae
Moraxellaceae
o__Chromatiales
Micrococcaceae
Microbacteriaceae
Leptotrichiaceae
Lactobacillaceae
Lachnospiraceae
Hyphomicrobiaceae
Helicobacteraceae
Gaiellaceae
Fusobacteriaceae
Erysipelotrichaceae
o__Acidimicrobiales
Enterobacteriaceae
Dethiosulfovibrionaceae
Desulfobulbaceae
Coriobacteriaceae
Comamonadaceae
Chitinophagaceae
Campylobacteraceae
Brucellaceae
Bradyrhizobiaceae
Bacteroidaceae
